# Need for hepatitis A prevention in patients with chronic liver disease in the changing epidemiological setting of India

**DOI:** 10.1080/21645515.2020.1832408

**Published:** 2020-11-25

**Authors:** Bhaskar Raju, Anar Andani, Shafi Kolhapure, Ashish Agrawal

**Affiliations:** a Dr Mehta’s Children’s Hospital, Chennai, India; b Global Medical Affairs, GSK, Wavre, Belgium; c Medical Affairs Department, GSK, Mumbai, India; d Medical Affairs Department, GSK, Hyderabad, India

**Keywords:** Hepatitis A, chronic liver disease, cirrhosis, India, vaccination, endemicity

## Abstract

The burden of chronic liver disease (CLD) in India is high, particularly among middle-aged men, with nearly 220,000 deaths due to cirrhosis in 2017. CLD increases the risk of infection, severe disease (e.g. hepatitis A virus or HAV superinfection, acute-on-chronic liver failure, fulminant hepatic failure), and mortality. Hence, various countries recommend HAV vaccination for CLD patients. While historic Indian studies showed high seroprevalences of protective HAV antibodies among Indian adults with CLD, the most recent ones found that nearly 7% of CLD patients were susceptible to HAV infection. Studies in healthy individuals have shown that HAV infection in childhood is decreasing in India, resulting in an increasing population of adults susceptible to HAV infection. As patients with CLD are at increased risk of severe HAV infection, now may be the time to recommend HAV vaccination among people with CLD in India.

## Chronic liver disease (CLD)

Hepatitis A, B, C, D, and E virus (HAV, HBV, HCV, HDV, and HEV) infections and alcohol consumption can cause liver damage, as can obesity, which can result in nonalcoholic fatty liver disease (NAFLD). CLD (disease that has lasted for ≥6 months) is a progressive deterioration in liver function, which can lead to fibrosis and cirrhosis.^1^ Cirrhosis often starts asymptomatically (“compensated cirrhosis”), but can ultimately progress to “decompensated cirrhosis”, during which complications of liver dysfunction and portal hypertension manifest (e.g. ascites, jaundice, variceal bleeding).^2^ Once a patient has decompensated cirrhosis, their survival will likely only be around 3–5 years.^3^ In 2017, there were an estimated 1.5 billion cases of cirrhosis and other CLDs globally.^4^ Liver cirrhosis was the 11^th^ and 26^th^ leading cause of disability-adjusted life years in men and women, respectively;^5^ the 13^th^ leading cause of life years lost;^6^ and, along with other CLDs, resulted in over 1.3 million deaths in 2017.^6^

Early treatment of patients with CLD is important. The goals of treatment are to stop disease progression (generally by managing the underlying cause, e.g. antivirals, alcohol abstinence) and to manage complications (e.g. portal hypertension, hepatorenal syndrome, bleeding esophageal varices, hepatic encephalopathy, and hepatocellular carcinoma).^1^ Ultimately, patients may require a liver transplant, which is the second commonest major organ transplantation.^7^

## Increased risk of severe infection

Patients with cirrhosis have a compromised immune system and are known to be at increased risk of bacterial infection,^8–12^ and those who become infected have a nearly 4-fold higher risk of death compared with uninfected people with cirrhosis.^2^ Given the effect of cirrhosis on the immune response, such patients may also be at increased risk of HAV infection. Although we could not find any confirmation of this, patients with CLD certainly appear to be at increased risk of developing more severe HAV disease if they have superimposed HAV disease.^13–15^ For example, in an outbreak of >300,000 HAV cases in China, mortality was 5.6-fold higher among those with HAV infection superimposed on underlying HBV infection than in those with HAV but without HBV.^15^ Acute HAV infection in patients with CLD can also result in acute-on-chronic liver failure (ACLF), which is associated with high rates of mortality.^16^

Patients with CLD are also at increased risk of developing fulminant hepatitis,^13,17^ also known as fulminant hepatic failure (FHF). In an Italian study, 595 adults (29.1 ± 9.8 years) with chronic HBV or HCV (without HAV antibodies) were enrolled during 1990–1997.^18^ Of these, 27 (4.5%) acquired HAV superinfection (10/163 of those with chronic HBV and 17/432 of those with chronic HCV). FHF developed in 0/10 chronic HBV patients and 7/17 chronic HCV patients, 6/7 of whom died. None of 191 controls (without CLD) who presented with acute HAV developed FHF.^18^ While this study implies that patients with chronic HBV are not at risk of FHF after HAV superinfection, results from a small Canadian study show that those with chronic HBV can have FHF after HAV superinfection. In the Canadian study, 4/60 cases of FHF during 1991–1997 were due to HAV.^19^ Three of these patients had CLD (2 chronic HBV infection; 1 alcoholic cirrhosis), and all 3 died (13–35 days after admission); the patient without CLD survived.^19^

## HAV vaccination recommendations in patients with CLD

The United States (US) Advisory Committee on Immunization Practices recommends a 2-dose series of HAV or a 3-dose series of HAV+HBV vaccinations for all patients with CLD, including those with HBV, HCV, cirrhosis, NAFLD, alcoholic liver disease, autoimmune hepatitis, or alanine aminotransferase or aspartate aminotransferase level >2 the upper limit of normal.^20^ Similarly, in the United Kingdom (UK), patients with various chronic liver conditions are recommended to receive HAV vaccination.^21^

Two types of HAV vaccine are available^22^ – live attenuated and inactivated – of which only the latter is appropriate for immunocompromised patients such as those with CLD. The World Health Organization (WHO) has endorsed that inactivated HAV vaccines are well tolerated by patients with mild-to-moderate CLD.^17^ It is recommended that HAV vaccination should be given as early as possible after CLD diagnosis for maximum efficacy and safety.^13,14^

## Situation in India

### Burden and changing etiology of CLD

In a multicenter prospective study conducted in different parts of India, 1.3% of nearly 21 million patients who attended 11 hospitals during February 2010 to January 2013 had liver disease.^23^ One quarter of these patients had a new diagnosis of liver disease, of whom 19.8% had CLD.^23^ Among these 13,014 patients with newly diagnosed CLD (of whom 4413 [33.9%] had decompensated cirrhosis), mean age was 42.8 ± 14.4 years and the majority (73.0%) were male.^23^ The main etiologies were related to hepatitis viruses (54.9%), alcohol (17.3%), and NAFLD (12.8%).^23^ However, etiology varied widely by region, with HCV being the most common in the North (44.9%), HBV in the East (47.9%) and South (40.5%), alcohol in the North-East (31.9%), and NAFLD in the Central region (43.6%) and the West (39.6%).^23^ CLD etiologies reported in other studies have varied widely,^24–34^ likely due to variations by region, population, and over time. The latter has been shown in a study in a tertiary care referral hospital in Eastern India, where etiologies of CLD changed substantially from 2003 to 2011, with alcohol increasing from 22.5% to 42.0% (*p* = .01), cryptogenic (i.e. unknown cause) decreasing from 44.9% to 25.0% (*p* = .001), but no significant changes in HBV (mean 22.3%) or HCV (mean 10.9%).^35^

More recent studies have indicated that NAFLD could be becoming a major cause of CLD in India, with huge numbers of people potentially affected. For example, a study published in 2016 found that 30.7% of adults aged ≥35 years in a rural community in North India had NAFLD on ultrasonography,^36^ while one published in 2019 reported that 528 (53.5%) of male blood donors (mean age 31 ± 8 years for males and 45 ± 8 for females) in an urban community in North India had NAFLD on ultrasonography.^37^

Recent meta-analyses have estimated seroprevalences of HBV surface antigen (HBsAg) and anti-HCV to be 1.46%^38^ and 0.44–0.88%,^39^ respectively in India. Based on a population of approximately 1.38 billion,^40^ this would equate to approximately 20 million people in India having chronic HBV infection and around 6–12 million having chronic HCV infection, meaning that large numbers of people are potentially at risk for FHF, which has a very high mortality rate among patients with CLD.^18,19^ However, these numbers are dwarfed by the potential number of people with NAFLD, which, based on the two above-mentioned studies^36,37^ and the adult Indian population,^40^ could equate to hundreds of millions of adults with NAFLD in India. While we were unable to find data on the prevalence of alcoholic liver disease in India, given that 18% of liver-related deaths in India were due to alcohol,^41^ there are likely also many millions of people with alcoholic liver disease in India.

### Mortality

In India, deaths due to cirrhosis nearly doubled – from 110,091 to 217,896 – between 1990 and 2017 (although there was little change in the age-standardized mortality rate).^42^ In 2017, 16.5% of global cirrhosis deaths (217,896 of 1,322,868) were in India.^42^

Mortality rates vary among patients with CLD. For example, those with alcoholic cirrhosis had higher 1-month mortality than those with nonalcoholic cirrhosis (9.8% vs. 3.2%) in a single-center study from North East India.^43^ In that study, patients with alcoholic versus nonalcoholic cirrhosis were more often male (97% vs. 64%) and had more advanced disease (based on various parameters).^43^ The rates of death or orthotopic liver transplantation within 1 year are even higher among those with a first episode of decompensation (most common presentations among 110 Indian patients with cirrhosis: overt ascites [57.3%], ultrasound-detected ascites [22.7%], and hepatic encephalopathy [13.6%]), occurring in 22.2%, 28.0%, and 20.0% of these patients, respectively.^33^ In an Indian retrospective study of 392 patients (median [range] age 50 [14–87] years; 80% male) who had died of liver-related causes (except liver metastasis from non-hepatic cancers), the most common causes of liver-related death were alcohol (30.1%), nonalcoholic steatohepatitis/cryptogenic (23.2%), hepatotropic viruses (18.6%), and bacterial/other infections (11.5%).^34^ Most patients (85.5%) had CLD, and among those with CLD, most (70.7%) had presented with cirrhosis complications (e.g. end-stage liver disease, portal hypertension, sepsis), while 29.3% presented with ACLF.^34^ Based on data from the WHO, approximately 54% of liver-related deaths in India are due to HBV, 18% due to alcohol, 10% due to HAV or HEV, and 10% due to HCV.^41^ However, acute hepatitis-related deaths are largely due to HBV (54%) or HEV (37%), with 6% due to HAV and 2% due to HCV.^41^

### ACLF

ACLF (acute decompensation in a patient with CLD^16^) occurred in 121/3220 (3.8%) patients with cirrhosis and acute HAV or HEV admitted to a hospital in Lucknow during 2000–2006.^32^ The mean age of those with ACLF was 36.3 ± 18.0 years and 70.2% were male.^32^ Clinical features included jaundice (100%), ascites (78.5%), coagulopathy (77.7%), encephalopathy (55.4%), hyponatremia (41.3%), renal failure (35.5%), and sepsis (33.9%).^32^ ACLF was due to HEV in 66.1%, HAV in 27.3%, or both in 6.6%; the underlying CLD was mainly cryptogenic (36.4%), HBV (30.6%), or alcohol (10.7%).^32^ Three-month mortality among these patients with ACLF was high (44.6%).^32^ In another retrospective study, which included 1049 consecutive patients with ACLF (mean age 44.7 ± 12.2 years; 81.3% male) conducted in 10 tertiary centers from across India during 2011–2014, the most common precipitants of ACLF were alcohol consumption (35.7%), viral superinfection/flare (HAV, HBV, or HEV) (21.4%), and sepsis (16.6%).^29^ The underlying CLD was mainly alcohol (56.7%), cryptogenic (19.4%), or HBV/HCV (15.9%). During a median (range) hospital stay of 8 (4–14) days, 42.6% of patients died.^29^ In a single-center study in Eastern India (2012–2014), the most common precipitants of acute decompensation among 123 patients with ACLF (mean age 45.8 ± 12.1 years; 88.6% male) were recent alcohol intake (42.3%) and bacterial infection (36.6%).^44^ Three-month mortality was very high (71.3%), more so in alcoholics than nonalcoholics (81.1% vs. 55.9%; *p* = .01).^44^ Lastly, among 64 patients (median age 44 years; 82.8% male) with ACLF in a hospital in Hyderabad, 2015–2016, the main precipitants were infection (43.8%) and alcoholism (37.5%). Twenty-eight day mortality was high (43.8%).^45^

### Susceptibility of Indian CLD patients to HAV

Nine out of ten old studies from India (carried out up to 2007)^25–28,30,31,46–48^ found that nearly all patients with CLD/cirrhosis (93.2–99.0%) had evidence of past infection with HAV (as shown by HAV-immunoglobulin G or HAV-IgG^49^ or anti-HAV antibodies), as did most healthy controls (71.2–100%) (Table 1). The study by Khanna et al.,^50^ however, reported a much lower rate of HAV-IgG among patients with cirrhosis (60.6%), possibly because they only included patients from the upper middle or upper socioeconomic classes. All of their seronegative CLD patients were vaccinated against HAV.^50^ The authors of most of the other studies suggested that CLD patients did not routinely require HAV vaccination (as most were already immune),^25,27,28,30,31,46–48^ while opinions on testing for HAV antibodies before potential vaccination were mixed (see Table 1).^25,31,48^

**Table 1.** Seroprevalence studies showing evidence of previous HAV infection (HAV-IgG or anti-HAV) in patients in India with CLD (or specifically with cirrhosis), listed chronologically

Reference	Study years	Population	HAV seroprevalence (%)	HAV vaccination recommendations for CLD patients	Other observations
Dhotial et al. 2002^46^	2000–2001	42 cirrhosis	97.6 (anti-HAV)	May not be needed in their population	Etiologies: alcohol (61.9%), HBV (16.7%), HCV (7.1%), other (14.3%)
Acharya et al. 2002^30^	1997–2001	254 CLD	97.6 (anti-HAV)	Not required	Etiologies: chronic hepatitis due to HCV (33.1%) or HBV (29.8%), cirrhosis due to HBV (18.5%) or HCV (18.5%)
Xavier and Anish 2003^31^	≤2002^a^	52 cirrhosis vs. 50 controls	98.1 vs. 100 (anti-HAV)	Routine vaccination not required; nor routine anti-HAV screening	Main etiologies: alcoholic cirrhosis (25.0%), HBV (13.5%); high/low socioeconomic class (51.9%/48.1%)
Ramachandran et al. 2004^27^	2001–2002	100 CLD vs. 79 controls^b^	99.0 vs. 100 (HAV-IgG)	Not needed	Main etiologies: cryptogenic (32.0%), alcohol (25.0%), HBV (25.0%)
Anand et al. 2004^28^	2002	187 CLD vs. 89 controls	95.7 vs. 94.6 (HAV-IgG)	Not routinely required	Main etiologies: HBV (48.7%), HCV (33.2%), cryptogenic (12.8%)
Duseja et al. 2004^47^	1999–2000	55 cirrhosis	98.2 (anti-HAV)	Routine vaccination cannot be recommended	Etiologies: alcohol (45.5%), HBV (23.6%), HCV (9.1%), Budd–Chiari Syndrome (3.6%), cryptogenic (20.0%)
Hussain et al. 2006^26^	1999–2003	300 CLD^c^ vs. 500 controls	98.0 vs. 71.2 (HAV-IgG)	Selective vaccination of high-risk population would be a rational and cost-effective approach	Etiologies: HBV (56.3%), HCV (24.3%), alcohol (16.0%), HBV+HCV (3.3%)
Khanna et al. 2006^50^	1999–2004	127 cirrhosis	60.6% (HAV-IgG)	All seronegative cirrhotic patients were vaccinated	All patients were upper/upper middle socioeconomic class
Joshi et al. 2007^25^	2004–2005	133 cirrhosis vs. 75 controls^b^	93.2 vs. 94.6 (anti-HAV)	Not routinely required screening for HAV antibodies may be more cost effective than vaccination	Main etiologies: HBV (29.3%), cryptogenic (23.3%), alcohol (21.8%), HCV (14.3%)
John et al. 2009^48^	≤2007^a^	300 CLD	93.3 (HAV-IgG)	Routine vaccination without testing for HAV antibodies not recommended	Etiologies: alcohol (62%), HBV/HCV (12%), cryptogenic (24%), Wilson’s (2%)

^a^These studies did not report study dates, so these dates are based on “received” and “accepted” publication dates.

^b^Age- and sex-matched.

^c^And HBV and/or HCV infection or alcoholism; CLD patients with unclassified etiology were excluded.

CLD, chronic liver disease; HAV, hepatitis A virus; HBV, hepatitis B virus; HCV, hepatitis C virus; IgG, immunoglobulin G; NR, not reported.

However, most of these studies are old, only including patients until 2007 at the latest and, as will be discussed further below, the epidemiology of HAV is changing in India, with declining HAV infection during childhood and subsequent increasing susceptibility in adulthood. It should also be noted that, in the two latest studies in Table 1,^25,48^ which included patients during the mid 2000s, nearly 7% of CLD patients did not have anti-HAV/anti-IgG antibodies and were therefore susceptible to HAV infection. As suggested by Radha Krishna et al. in 2009^32^ – based on their study that included 21 adults with ACLF due to HAV – this advice may now be outdated. In a more recent study (2011–2014), 21.4% of ACLF cases were due to HAV, HBV, or HEV superinfection, but unfortunately, the authors did not report results separately for HAV.^29^

### Changing HAV endemicity in India

If HAV is encountered during early childhood, the resultant hepatitis is generally mild, causing no symptoms or nonspecific symptoms (e.g. fever, malaise, diarrhea)^51^ and providing long-term immunity against HAV.^52^ However, if HAV is encountered for the first time in adulthood, most people will have symptoms (e.g. jaundice, pain), and it is associated with a mortality rate of around 1%.^51^ HAV is also significantly more likely to result in more severe disease with increasing age.^53^ Historically, many people in India were exposed to HAV during childhood, resulting in life-long protection.^52^ However, with improved sanitation and hygiene, children are becoming less likely to be exposed to HAV, resulting in increasing number of adolescents and adults who are at risk of infection, and a paradoxical increase in morbidity and mortality due to HAV.^51,52,54,55^ Thus, and taking into account the high heterogeneity across the Indian continent, India is now thought to be shifting from high to intermediate HAV endemicity.^51,54^ This situation is particularly challenging, as in high HAV endemicity areas, most children are exposed, resulting in mild disease and lifelong immunity, while in low endemicity areas, the chance of exposure in adulthood is low.^54^ However, with intermediate HAV endemicity, the chance of childhood exposure is reduced, leaving more adults at risk of more severe disease.^54^

### Declining HAV immunity in India

**Figure 1.**
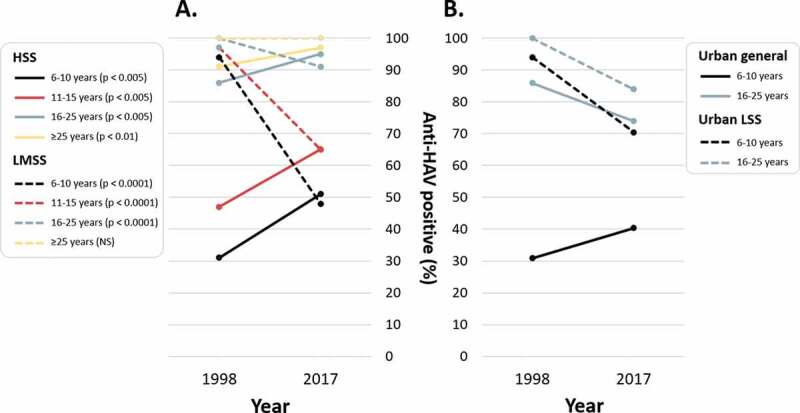
Opposing, but converging, trends in HAV susceptibility among (A) higher and lower middle socioeconomic status populations (created based on data from Arankalle et al.^59^) and (B) urban general and lower socioeconomic status populations (created based on data from Deoshatwar et al.^61^)

HAV, hepatitis A virus; HSS, high socioeconomic status; LMSS, lower middle socioeconomic status; LSS, lower socioeconomic status; NS, not significant; *p, p* value.

Various serological studies have reported that the proportion of healthy Indian people with seroprotective anti-HAV antibodies (i.e. previous HAV infection) has fallen over time.^26,56–59^ For example, Arankalle et al.^56^ reported that anti-HAV positivity decreased significantly from 1982 to 1998 among children from urban high socioeconomic populations (age 6–10 years: ~86% to 30.9%; age 11–15 years: ~94% to 46.9%; combined age *p* < .00001), but not in adults or urban lower middle socioeconomic populations. Das et al.^57^ reported that HAV-IgG seropositivity fell from 98.0% in 1982^60^ to 54.1% in 1998 among those aged 15–24 years and from 98.6%^60^ to 58.7% among those aged 25–34 years (both *p <* .05). Hussain et al.^26^ reported that 71.2% of healthy subjects were positive for HAV-IgG in 1999–2003, much lower than the 94.8% reported in subjects in 1982 in an earlier study.^60^ Gadgil et al.^58^ found that HAV seropositivity among adult blood donors fell from 96.5% in 2002 to 92.1% in 2004–2005 (*p* < .01). Recently, Arankalle et al.^59^ reported that, while HAV seropositivity decreased from 1998 to 2017 among low/middle socioeconomic children and younger adults, it increased during the same time period among high socioeconomic children and adults (Figure 1A).^59^ This was likely due to HAV vaccination in the high socioeconomic population, although the vaccination status of the participants was not available. Figure 1A also shows that, in 1998, low/middle socioeconomic populations had considerably higher seropositivity (i.e. were much more likely to have had previous HAV infection) than high socioeconomic populations of the same age group, but by 2017, there was very little difference in seropositivity between the two populations.^59^ Deoshatwar et al.^61^ have also reported results from the same region (for select age groups) for 2017 and compared them with the same 1998 data as Arankalle et al.^59^ Figure 1B shows that the changes in seropositivity were less pronounced in the latter study.

Arankalle et al.^59^ also reported that 90–95% of 3-month-old infants in both 1995 and 2017 were seropositive for HAV, likely due to maternal antibodies. In 1995, seropositivity fell to 13.6% by age 9 months and then increased to 41.0% by age 15 months, which must have been due to natural infection as none were vaccinated. However, in 2017, seropositivity fell to only 2.2% among unvaccinated infants at age 15 months.^59^ These studies all support a decrease in natural HAV infection during childhood, resulting in an increase in the number of susceptible adults.

### Increasing HAV infection in adulthood

**Figure 2.**
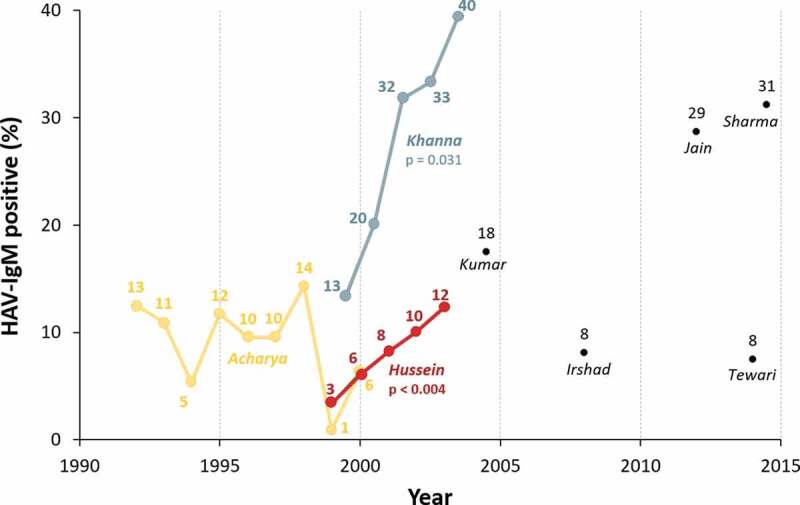
Increasing acute HAV infection among adults with acute viral hepatitis, created based on data from Acharya et al. 2003,^62^ Khanna et al. 2006 (middle adult age group used),^50^ Hussain et al. 2006,^26^ Kumar et al. 2007 (mainly adults),^63^ Irshad et al. 2010,^64^ Jain et al. 2013,^65^ Tewari et al. 2016,^66^ and Sharma et al. 2016 (suspected viral hepatitis).^67^ Further information on these studies can be found in Table 2

HAV, hepatitis A virus; IgM, immunoglobulin M.

In line with declining HAV seroprotection, some studies have shown an increase in the proportion of acute viral hepatitis cases that are due to HAV over time.^26,50^ Hussain et al.^26^ studied 1932 patients with acute viral hepatitis at a tertiary care center in Northern India, of whom 11.4% overall were HAV-IgM positive (indicating current infection). This increased from 3.4% in 1999 to 12.3% in 2003 among adults (Hussain line in Figure 2; *p* < .004); and from 10.6% to 22.0% in children (*p* < .003). At another tertiary care center in Northern India, Khanna et al.^50^ reported increasing proportions of acute hepatitis due to HAV from 1999 to 2004 among patients with acute hepatitis aged 13–20 years (27.2% to 61.5%; *p* = .008), 21–30 years (13.3% to 39.5%; Khanna line in Figure 2; *p* = .031), and >30 years (0% to 17.3%; *p* = .06).

Various other Indian studies have reported on the seroprevalence of HAV-IgM antibodies among those with acute viral hepatitis, but have reported no change over time (Acharya line in Figure 2),^62^ or have not studied their evolution over time^63-69^ (see Table 2 and single points in Figure 2). While comparisons between studies (particularly single-center studies) should be undertaken with caution, as seropositivity varies widely by socioeconomic status, age, HAV vaccination rates, region, setting, and local outbreaks, there appears to be a slight upward trend in the proportion of adults with acute viral hepatitis who have acute HAV infection (Figure 2).

**Table 2.** Seroprevalence studies showing evidence of current HAV infection in patients in India with (suspected) acute viral hepatitis, listed chronologically

Reference	Study years	Population	Age	Seroprevalence (HAV-IgM) (%)	Other observations
Poddar et al. 2002^68^	1997–2000	172 AVH	Children	72.7 (64.5 HAV alone; 8.1 with HCV and/or HEV)	Other etiologies: HEV 16.3%, HBV 7.6%
Acharya et al. 2003^62^	1992–2001	998 AVH vs. 492 FHF	Adults	7.7 vs 5.9	No significant change in the proportion of AVH or FHF due to HAV from 1992 to 2001
Hussain et al. 2006^26^	1999–2003	1932 AVH	751 children, 1181 adults	16.2 (children), 8.4 (adults)	See line in Figure 2 for evolution over time
Khanna et al. 2006^50^	1999–2004	500 AVH	90 children, 410 adults	72.2 (children), 28.0 (adults)	All middle/upper socioeconomic status; see line in Figure 2 for evolution over time
Kumar et al. 2007^63^	2002–2006	685 AVH vs. 70 FHF vs. 53 CLD vs. 11 ATT-induced jaundice vs. 24 pregnant	10–70 years	17.5 vs. 4.3 vs. 0 vs. 0 vs. 0	–
Irshad et al. 2010^64^	2006–2009	76 AVH vs. 54 FHF vs. 102 CVH vs. 96 cirrhosis vs 42 HCC	Adults	8.1 vs. 0 vs. 0 vs. 0 vs. 0	–
Jain et al. 2013^65^	2011–2012	205 AVH vs. 62 FHF	AVH: 97 children, 108 adults; FHF: 46 children, 16 adults	AVH: 34.0 (children), 28.7 (adults); FHF: 13.0 (children), 12.5 (adults)	Other etiologies (AVH and FHF combined): HEV 18.0%, HBV 16.1%, HCV 12.0%
Sharma et al. 2016^67^	2012–2015	285 suspected viral hepatitis	Adults	36.8 (31.2 HAV alone; 5.6 with HBV, HCV, or HEV) ^a^	Other etiologies: HBV 1.8%, HCV 1.4%, HEV 1.4%
Tewari et al. 2016^66^	2014	89 AVH	36 children, 53 adults	72.2 (children), 7.5 (adults)	–
Mittal et al. 2016^69^	2015	1654 AVH	Children and adults	7.7	Most seropositive cases were aged 11–20 years (45.6%), 0–10 years (29.1%), 21–30 years (18.1%)

AVH, acute viral hepatitis; ATT, antituberculosis treatment; CVH, chronic viral hepatitis; CLD, chronic liver disease; FHF, fulminant hepatic failure; HAV, hepatitis A virus; HBV, hepatitis B virus; HCC, hepatocellular carcinoma; HCV, hepatitis C virus; HEV, hepatitis E virus; IgM, immunoglobulin M.

The seroprevalence among children varied widely by study, from 16.2%^26^ to 72.2%,^50,66^ with little correlation over time. This may relate to the socioeconomic status of the studied populations (which, as shown in Figure 1, used to have a large impact on seroprevalence, but nowadays, has much less impact), but most studies did not describe this parameter.

### Indian HAV immunization recommendations

HAV vaccination is not included in the routine childhood immunization schedule in India.^70^ However, the Advisory Committee on Vaccines & Immunization Practices (ACVIP) of the Indian Academy of Pediatrics (IAP) recommends HAV vaccination for all infants, as a single dose of live attenuated vaccine at 12 months or 2 doses of inactivated vaccine at 12 and 18 months of age,^51^ which can be administered in a private setting paid for by the parents.^22^ The IAP particularly recommends HAV vaccination for various risk groups, including children with CLD and those who are carriers of HBV and HCV.^51^

Although the recommendation to vaccinate patients with CLD against HAV has been endorsed by the WHO,^17^ the Indian National Centre for Disease Control (NCDC) does not currently recommend HAV vaccination for adults with CLD in India, as “most adults have already been exposed to and are thus protected”.^71^ This recommendation is supported by 9/10 old studies from India (carried out up to 2007)^25–28,30,31,46–48^ (Table 1). However, in the current context of changing endemicity it is very unlikely to hold true and therefore we feel that this should now be reexamined.

Indian associations and scientific society recommendations relating to HAV vaccination are detailed in Table 3.^72–74^ The Association of Physicians of India (API) and the Indian Society of Nephrology (ISN) both indicate that patients with CLD who are not immune to HAV, those with other hepatitis virus infections, and patients awaiting or having received a liver transplant are at risk of HAV infection, but do not specifically recommend vaccination.^72,73^ The ISN, however, says that HAV vaccination “is indicated for all transplant candidates with CLD or those patients of end‑stage renal disease who have chronic hepatitis B or C” due to an increased risk of FHF.^73^ The Indian Medical Association (IMA) does not mention CLD or other hepatitis infection, but does recommend HAV for adults or children undergoing liver transplantation.^74^

**Table 3.** Adult HAV vaccination guidelines from various associations and scientific societies in India

	API 2009^72^	ISN 2016^73^	IMA 2018^74^
All adults	No	No	Yes
CLD and not immune to HAV	Unclear^a^	Unclear^a^	NM
Other hepatitis viruses	Unclear^a^	Unclear^a^	NM
Liver transplantation	Unclear^a^	Unclear^a^	Yes
Transplant candidates with CLD	NM	Yes	NM
ESRD and chronic HBV or HCV	NM	Yes	NM
Other at-risk people^b^	Unclear^a^	Unclear^a^	Yes

^a^Guideline specifies that these people are at high risk for acquiring HAV, and are most likely to benefit from HAV vaccination, but it is not clear whether vaccination is recommended.

^b^Definitions vary by guideline.

API, Association of Physicians of India; CLD, chronic liver disease; ESRD, end-stage renal disease; HAV, hepatitis A virus; HBV, hepatitis B virus; HCV, hepatitis C virus; IMA, Indian Medical Association; ISN, Indian Society of Nephrology; NM, not mentioned.

## Authors’ recommendations

Based on the currently available evidence of shifting endemicity and increasing HAV susceptibility in adulthood in India, now may be the time to revisit the existing NCDC recommendation that HAV vaccination is not necessary for those with CLD in India.^71^ Instead, we propose that a recommendation for HAV vaccination of adults with CLD should be considered in India, as is already the case in the US,^20^ the UK,^21^ and Sri Lanka^75^ (a near neighbor of India), and also for children with CLD in India.^51^ While some Indian medical association/society guidelines recognize that seronegative patients with CLD are at increased risk of HAV infection, they do not clearly recommend HAV vaccination.^72,73^

Up-to-date serological studies among Indian patients with CLD would be beneficial to confirm whether seroprotective HAV antibodies have decreased over time in these patients, in line with what has been shown in healthy people.^26,56–59,61^ However, awaiting the results of such studies should not be a prerequisite for recommending HAV vaccination among patients with CLD in India.

A more targeted approach, with serological testing prior to HAV vaccination, could be a more cost-effective option than universal HAV vaccination of patients with CLD.^25^ However, given the limited facilities for serological testing, the associated cost, and the potential for missed opportunity for vaccination if patients do not return after serological testing, this should also not be a prerequisite.

### Limitations

This was not a systematic review, so although we included all relevant manuscripts that we could find on PubMed and Embase, there may have been some manuscripts (e.g. those published in Indian journals that are not listed on PubMed or Embase) that we did not manage to capture. Also, India is a large country with high socioeconomic status heterogeneity. As seropositivity rates vary considerably with socioeconomic status, age, HAV vaccination rates, region, setting, and local outbreaks, comparisons between studies from different time periods should be undertaken with caution. Futher, as already discussed, the studies that have assessed the HAV susceptibility of CLD patients are old and, while it is likely that susceptibility among these patients has increased as it has among general adults, this should be confirmed.

## Summary and conclusions

The burden of CLD in India is high, resulting in high morbidity and mortality.^42^ Patients with CLD are at increased risk of severe HAV disease^13-15,17^ and ACLF, which has a very high mortality rate.^16,29,32,44,45^ Hence, such patients are recommended to receive various vaccinations, including HAV vaccination, in the US,^20^ the UK,^21^ and Sri Lanka.^75^ Old studies from India showed a high seroprevalence of protective HAV antibodies among Indian adults with CLD,^25–28,30,31,46–48^ although the most recent ones (≤2007) found that nearly 7% of CLD patients did not have protective HAV antibodies and were therefore susceptible to HAV infection. Studies in healthy individuals have shown that HAV infection in childhood is decreasing in India,^56–59,76^ resulting in an increasing population of adults without protective antibodies, and a higher risk of HAV infection in adulthood.^26,50^ This is likely also the case among patients with CLD.

Based on seroprovalence data,^38,39^ millions of people in India likely have chronic HBV or HCV infection. Even more adults could have NAFLD^36,37^ and, along with an increasing amount of alcoholic liver disease in India,^35^ this equates to a huge population of people with chronic hepatitis infection and/or CLD. Such people are at higher risk of severe disease^13,17^ (HAV superinfection, ACLF, FHF) and increased mortality.^15,18,19^

It may, therefore, now be time to reexamine the existing Indian recommendations.^71–74^ Patients with CLD who do not have HAV antibodies should receive HAV vaccination. This approach could reduce morbidity, mortality, and healthcare costs of HAV infection among patients with CLD.^77^ In situations where antibody testing is not available or practical, CLD patients should not be excluded from HAV vaccination.

**Figure 3.**
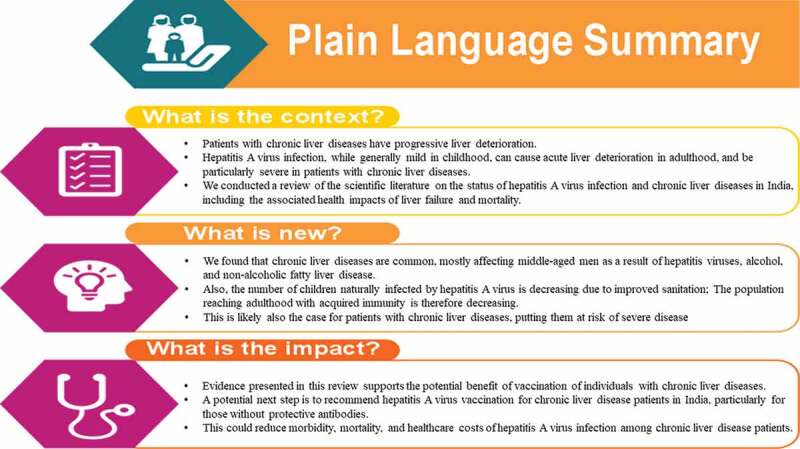
Plain language summary

Figure 3 elaborates on the findings in a form that could be shared with patients by healthcare professionals.
